# Supplementary material for: Activation of GPR3-β-arrestin2-PKM2 pathway in Kupffer cells stimulates glycolysis and inhibits obesity and liver pathogenesis
Source: Nat Commun. 2024 Jan 27;15:807. doi: 10.1038/s41467-024-45167-5 (PMC10821868; doi:10.1038/s41467-024-45167-5)
Supplement: Supplementary file 1 — Supplementary Information [file 41467_2024_45167_MOESM1_ESM.pdf]

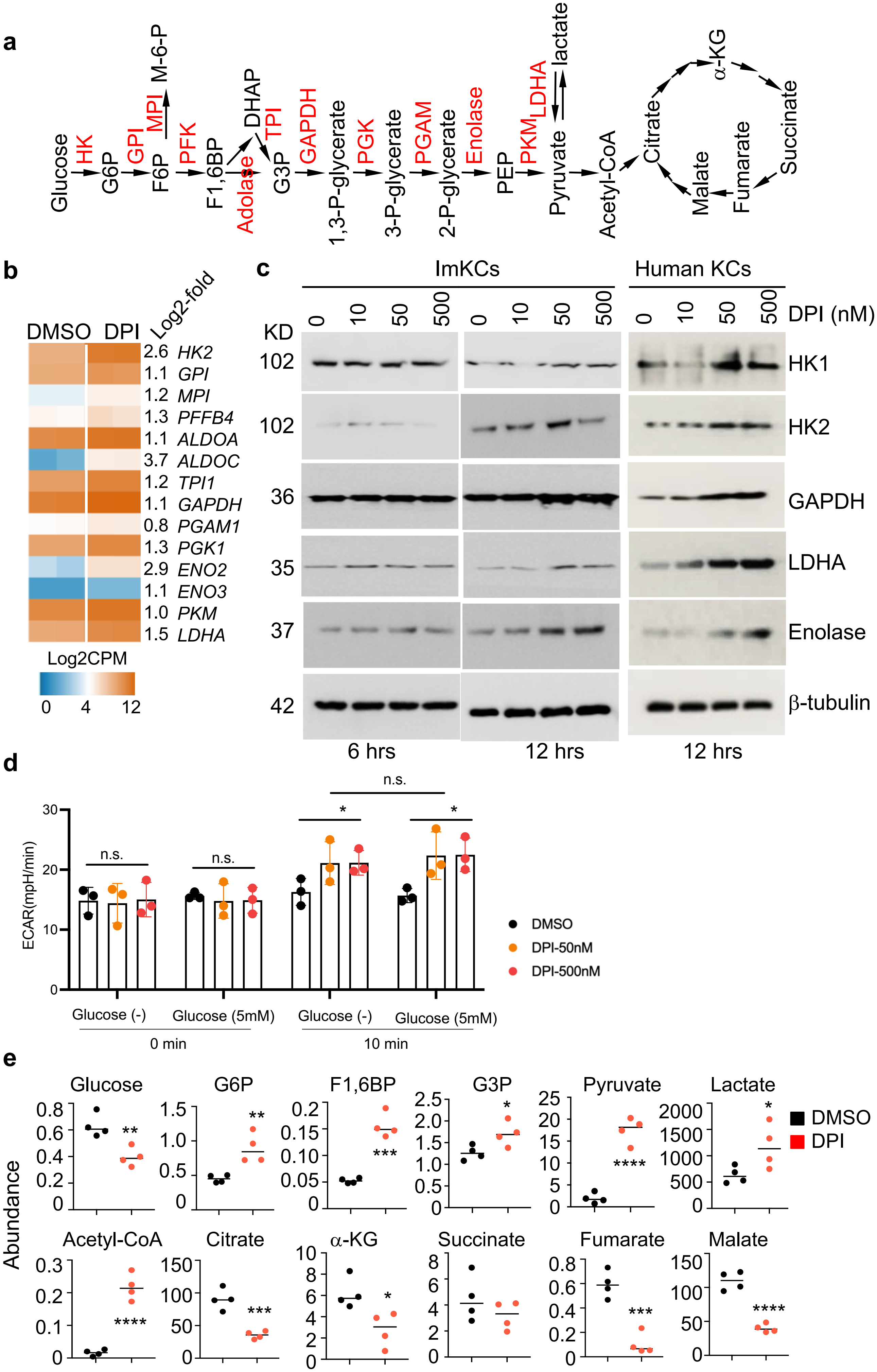
a
Glucose
HK
G6P
GPI
F6P
MPI
M-6-P
PFK
F1,6BP
Adolase
DHAP
TPI
G3P
GAPDH
1,3-P-glycerate
PGK
3-P-glycerate
PGAM
2-P-glycerate
Enolase
PEP
PKM
LDHA
lactate
Pyruvate
Acetyl-CoA
Citrate
α-KG
Succinate
Fumarate
Malate
b
DMSO
DPI
Log2-fold
2.6 HK2
1.1 GPI
1.2 MPI
1.3 PFFB4
1.1 ALDOA
3.7 ALDOC
1.2 TPI1
1.1 GAPDH
0.8 PGAM1
1.3 PGK1
2.9 ENO2
1.1 ENO3
1.0 PKM
1.5 LDHA
Log2CPM
0 4 12
c
ImKCs
Human KCs
KD
0 10 50 500 0 10 50 500 0 10 50 500 DPI (nM)
102 HK1
102 HK2
36 GAPDH
35 LDHA
37 Enolase
42 β-tubulin
6 hrs
12 hrs
12 hrs
d
ECAR(mpH/min)
30
20
10
0
n.s.
n.s.
*
*
n.s.
Glucose (-)
Glucose (5mM)
Glucose (-)
Glucose (5mM)
0 min
10 min
DMSO
DPI-50nM
DPI-500nM
e
Abundance
Glucose
G6P
F1,6BP
G3P
Pyruvate
Lactate
DMSO
DPI
Acetyl-CoA
Citrate
α-KG
Succinate
Fumarate
Malate

Supplementary Fig. 1. DPI stimulates both rapid and sustained increase in glycolysis in macrophages. **a**, Glycolysis pathway with involved enzymes and intermediates and TCA cycle with selected intermediates. **b**. DPI stimulates transcription of glycolytic genes in human primary macrophages following treatment with 50 nM DPI for 24 hours. Heatmap of transcript levels is based on reanalysis of RNAseq data from Hu et al. 2021. **c**. DPI stimulates expression of glycolytic enzymes at protein level as measured by Western blotting. Total protein lysates were isolated from either mouse ImKCs with or without DPI treatment for 6 and 12 hrs or human primary KCs (AcceGen) with or without DPI treatment for 12 hrs at the indicated DPI concentrations. Equal amounts of total proteins from whole-cell lysates were subjected to Western blotting analysis. β-actin was used as a loading control. Shown are representative data of two independent experiments. **d**. DPI immediately stimulated ECAR (glycolysis) with or without extracellular glucose (5mM). ECAR was measured for seeded ImKCs at 0 min and at 10 min following the addition of DPI (50nM or 500nM). Shown are representative data of three independent experiments. **e**. Metabolite analysis in ImKCs. ImKCs were treated with DPI (500 nM) for 24 hrs and the select metabolites were quantified by LC-MS. Shown are representative data of two independent experiments. P values were calculated by the student's t-test. * P <0.05, ** P <0.01, *** P <0.001.. n.s. not significant.

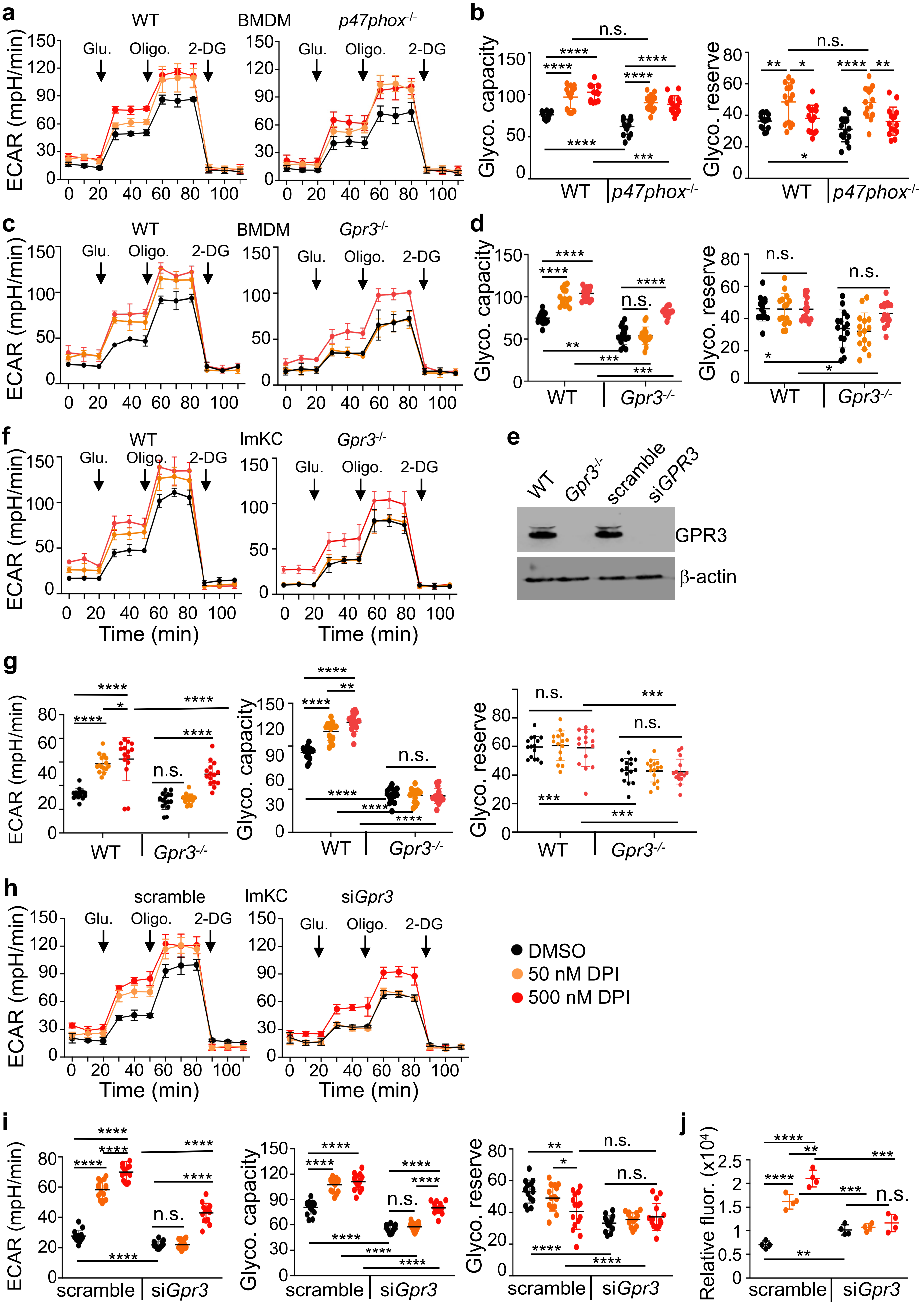
a
WT
BMDM
p47phox-/-
Glu.
Oligo.
2-DG
ECAR (mpH/min)
b
Glyco. capacity
Glyco. reserve
n.s.
WT
p47phox-/-
c
WT
BMDM
Gpr3-/-
d
e
WT
Gpr3-/-
scramble
siGPR3
GPR3
β-actin
f
WT
ImKC
Gpr3-/-
Time (min)
g
h
scramble
ImKC
siGpr3
DMSO
50 nM DPI
500 nM DPI
i
j
Relative fluor. (x10^4)

k

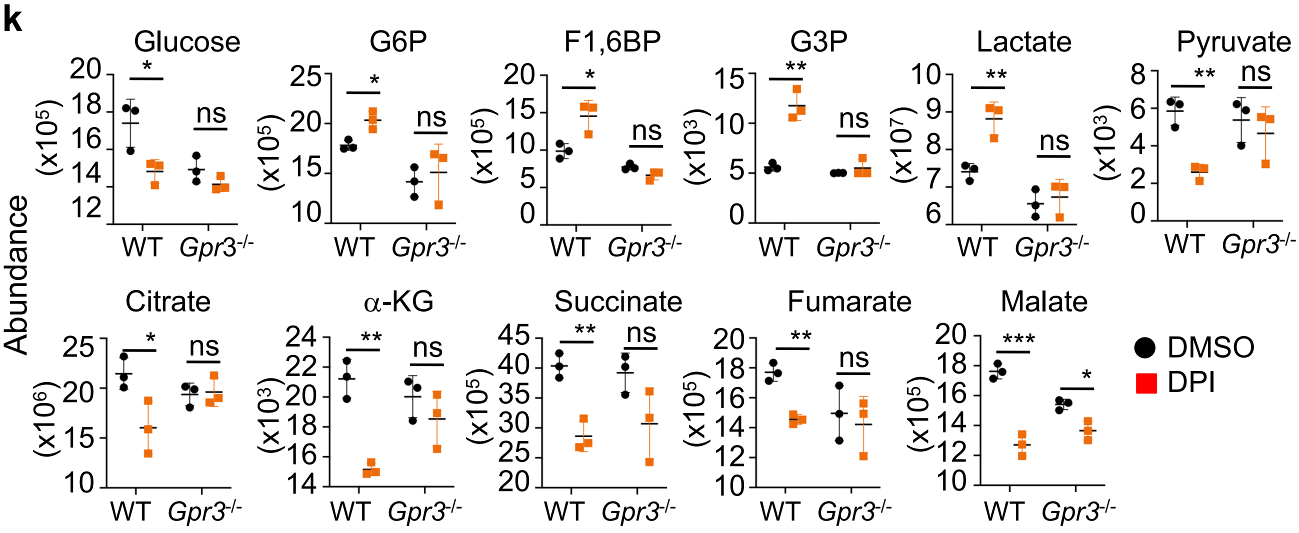

Supplementary Fig. 2. DPI stimulates glycolysis through GPR3. **a-b**. DPI-stimulated glycolysis is independent of the NOX activity. Wildtype (WT) and *p47phox*$^{-/-}$ BMDMs were seeded and incubated without or with DPI (50 and 500 nM) for 24 hrs and ECAR was measured by Seahorse analyzer. Specific parameters for glycolytic capacity and glycolytic reserve were calculated and summarized based on two independent experiments. Data are presented as the mean ± sd from three independent experiments (n=15 biological replicates). **c-d.** Comparison of DPI's effect on glycolysis in wildtype and *Gpr3*$^{-/-}$ BMDMs. BMDMs were generated from wildtype and *Gpr3*$^{-/-}$ mice, seeded and incubated with or without DPI (50 and 500 nM) for 24 hrs and ECAR was measured by Seahorse analyzer. Data are presented as the mean ± sd from three independent experiments (n=15 biological replicates). **e**. Western blotting of WT and *Gpr3*$^{-/-}$ ImKCs, and ImKCs transfected with siRNA specific for *Gpr3* (siGpr3) or scrambled siRNA. **f-g**. Comparison of DPI's effect on glycolysis in wildtype and *Gpr3*$^{-/-}$ ImKCs. *Gpr3*$^{-/-}$ ImKC were constructed by CRISPR-Cas9-mediated gene editing. WT and *Gpr3*$^{-/-}$ ImKCs were seeded and incubated with or without DPI (50 and 500 nM) for 24 hrs and ECAR was measured by Seahorse analyzer. Data are presented as the mean ± sd from three independent experiments (n=15 biological replicates). **h-i**, ImKCs were transfected with siGpr3 or scrambled siRNA. 48 hours later, transfected ImKCs were seeded and incubated without or with DPI (50 and 500 nM) for 24 hrs and ECAR was measured by Seahorse analyzer. Data are presented as the mean ± sd from three independent experiments (n=15 biological replicates). **j.** The effect of DPI on glucose uptake in ImKCs. Scramble and *siGPR3* ImKCs were treated with DMSO or DPI (50 and 500 nM) for 24 hrs in the presence of the fluorescent glucose analog 2-NBDG. The mean fluorescence intensity (MFI) of 2-NBDG in cells was measured by flow cytometry and normalized to DMSO controls. Data are presented as the mean ± sd (n=4). **k**. Metabolite analysis in WT and *Gpr3*$^{-/-}$ ImKCs. WT and *Gpr3*$^{-/-}$ ImKCs were treated with DPI (50 nM) for 24 hrs and the select metabolites were quantified by LC-MS. Data are presented as the mean ± sd from n=3 independent experiments. P values were calculated by student t-test. * P <0.05, ** P <0.01, *** P <0.001, **** P<0.0001. n.s. not significant.

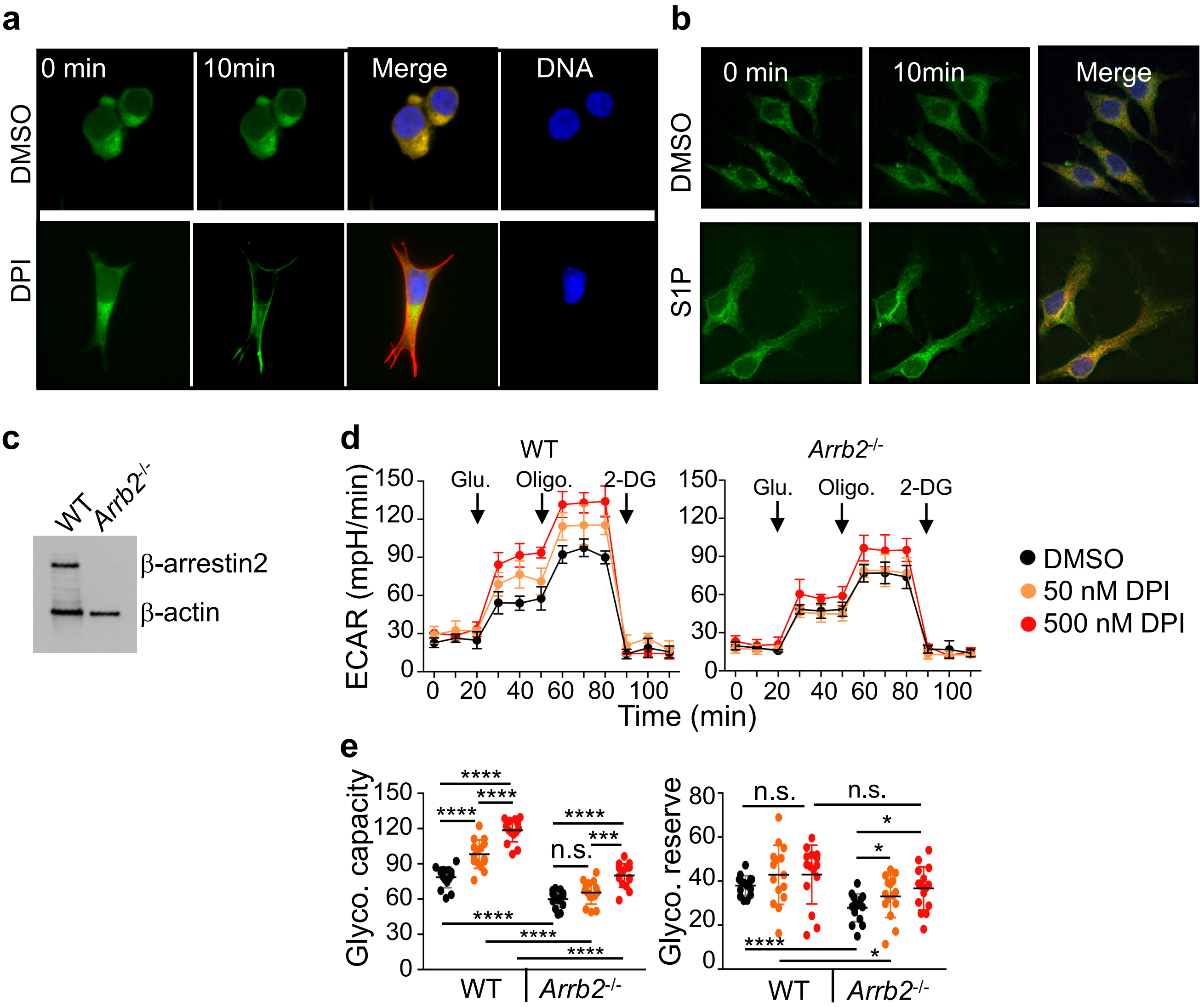

Supplementary Fig. 3. DPI stimulates glycolysis through β-arrestin2. **a-b**. BMDMs were transfected with *Arrb2-GFP* fusion gene and stimulated with DMSO or DPI (50 nM) (**a**) or S1P (3 mM) (**b**). The GFP signal was captured with a TIRF microscope at indicated time points. Shown are representative data of GFP (green) signal at 0 min and 10 min, and the merged signal (green for 0 min and red for 10 min) from three independent experiments. **c**. Western blotting of β-arrestin2 in wildtype or *Arrb2*$^{-/-}$ ImKCs. **d-e**. WT and *Arrb2*$^{-/-}$ ImKCs were seeded and incubated without or with DPI (50 and 500 nM) for 24 hrs and ECAR was measured by Seahorse analyzer. Data are presented as the mean ± sd from three independent experiments (n=15 biological replicates). P values were calculated by student t-test. * P <0.05, ** P <0.01, *** P <0.001, **** P<0.0001. n.s. not significant.

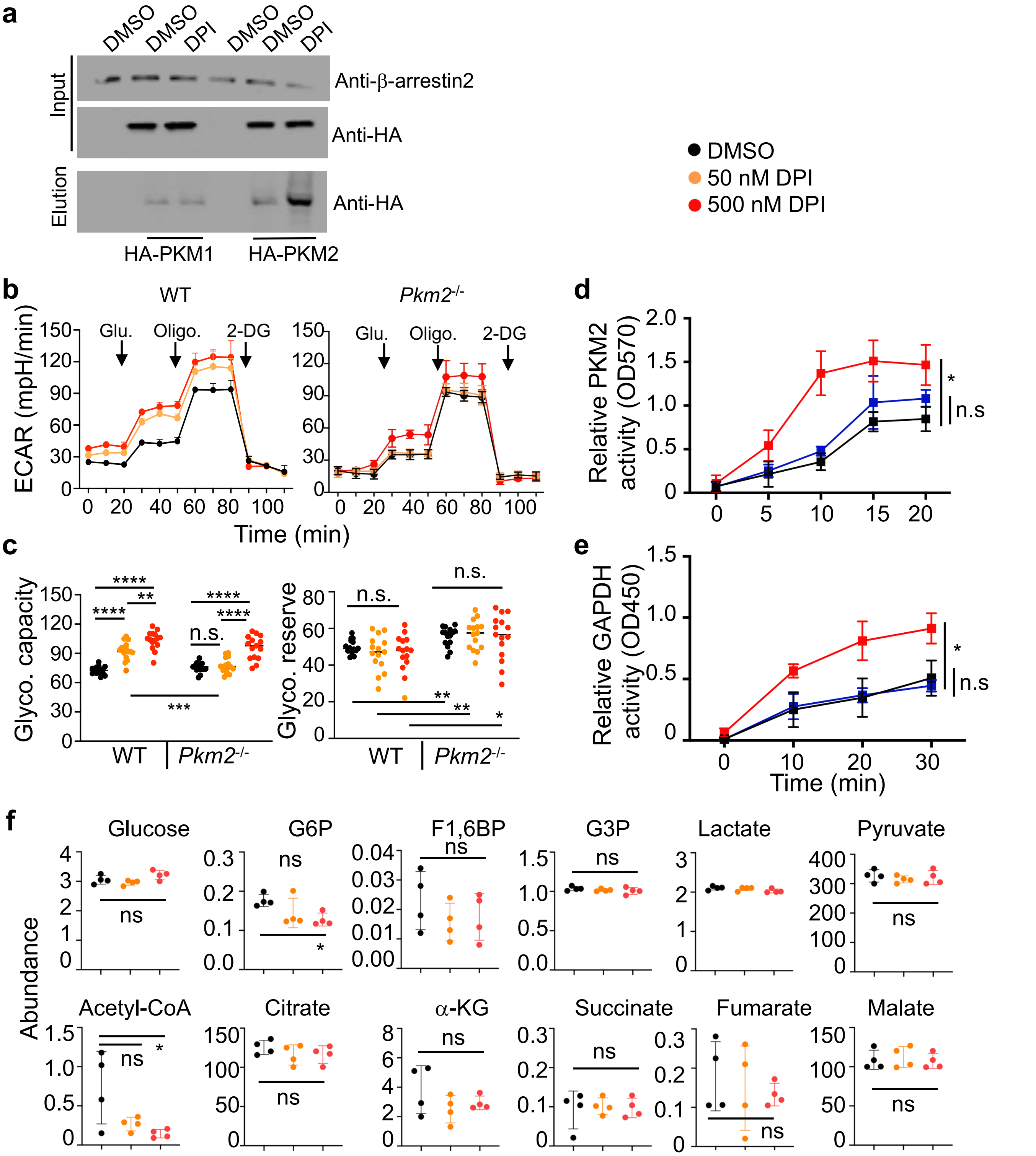

Supplementary Fig. 4. DPI stimulates glycolytic activity through PKM2. a. Co-immunoprecipitation of β-arrestin2 with PKM1 or PKM2. ImKCs were co-transfected with β-arrestin2 and either HA tagged PKM1 or PKM2 and 48h later were treated with or without 50 nM DPI for 6 hrs. Cell lysates were immunoprecipitated with anti-β-arrestin2 and the precipitates were analyzed by Western blotting for the indicated proteins. Shown are representative data from one of the two independent experiments. **b-c.** Comparison of DPI's effect on glycolysis in wildtype and *Pkm2*$^{-/-}$ BMDMs. BMDMs were generated from wildtype and *Pkm2*$^{-/-}$ mice, treated with or without DPI (50 and 500 nM) for 24 hrs and ECAR was measured by Seahorse analyzer. Data are presented as the mean ± sd from three independent experiments (n=15 biological replicates). **d-e.** Activation of PKM2 and GAPDH enzymatic activity by DPI is inhibited by ERK1/2 inhibitor. ImKCs were treated with DMSO (black line) or DPI alone (500 nM) (red line) or DPI plus ERK1/2 inhibitor (SCH772984, 1μM) (blue line) for 6 hrs and the enzymatic activities of PKM2 (d) and GAPDH (e) were measured by colorimetric assay kits (Biovision). Data are presented as the mean ± sd (n=6). **f**. Metabolite analysis in *Pkm2*$^{-/-}$ mouse BMDMs. *Pkm2*$^{-/-}$ BMDMs were treated with DMSO or DPI (50 nM and 500 nM) for 24 hrs and the select metabolites were quantified by LC-MS (n=4). P values were calculated by the student's t-test. * P <0.05, ** P <0.01, *** P <0.001, **** P<0.0001. n.s. not significant.

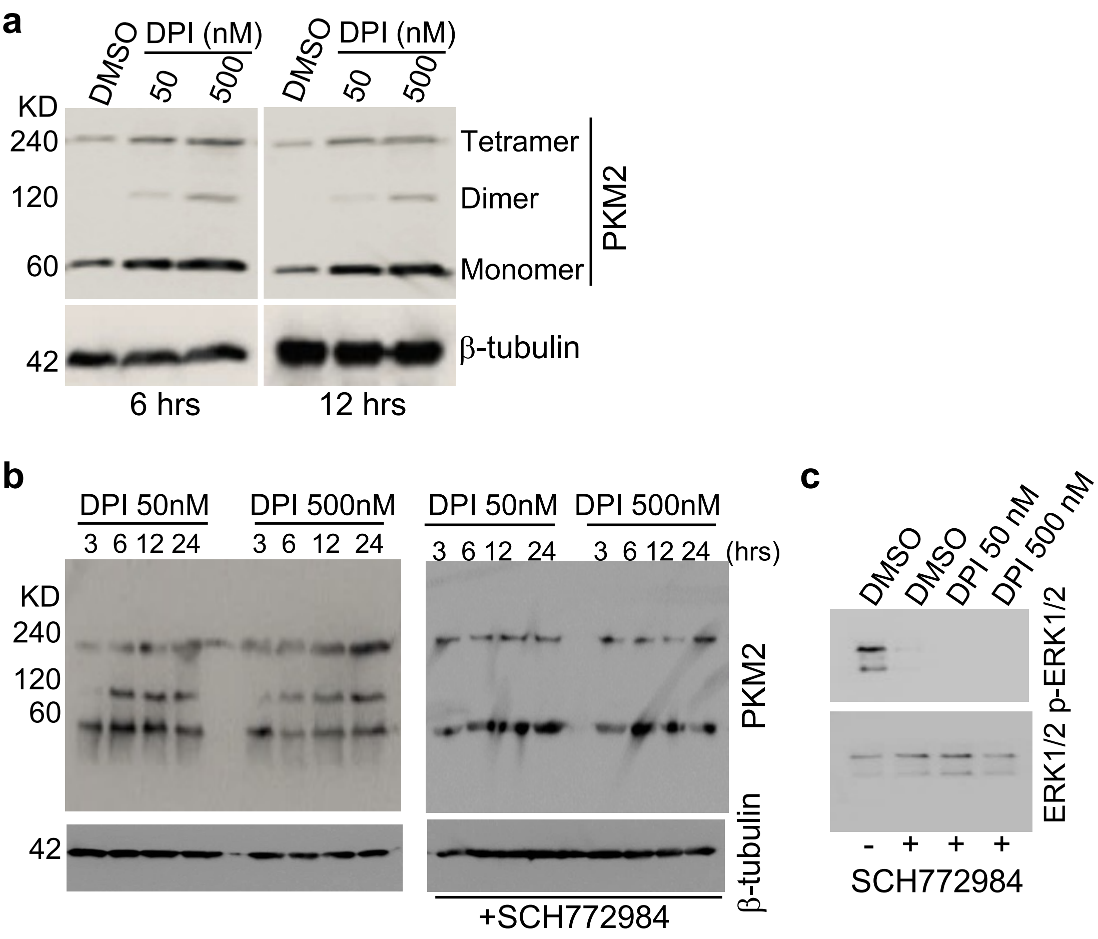

Supplementary Fig. 5. DPI stimulates a sustained increase in glycolytic activity through nuclear translocation of PKM2 and transcriptional activation of c-Myc.

a. Induction of dimeric PKM2 by DPI. ImKCs were treated with vehicle or DPI (50 and 500 nM) for 6 or 12 hrs. Cell lysates were run on native PAGE gel and analyzed by Western blotting. Shown are representative data from two independent experiments. **b.** Dimeric PKM2 was abolished by ERK1/2 inhibitor. ImKCs were treated with DPI (50 and 500 nM) for 3, 6, 12 or 24 hrs in the absence or presence of SCH772984. Cells were then treated with crosslinking agent DSS and lysed. Lysates were run on SDS-PAGE and analyzed by Western blotting. Shown are representative data from two independent experiments. **c**. Phosphorylation of ERK1/2 is inhibited by SCH772984 in the presence of DPI. ImKCs were treated with vehicle or DPI (50 and 500 nM) in the presence or the absence of SCH772984 for 12 hrs. Cells were lysed and analyzed for total ERK1/2 and phosphorylated ERK1/2 by Western blotting. Shown are representative data from two independent experiments.

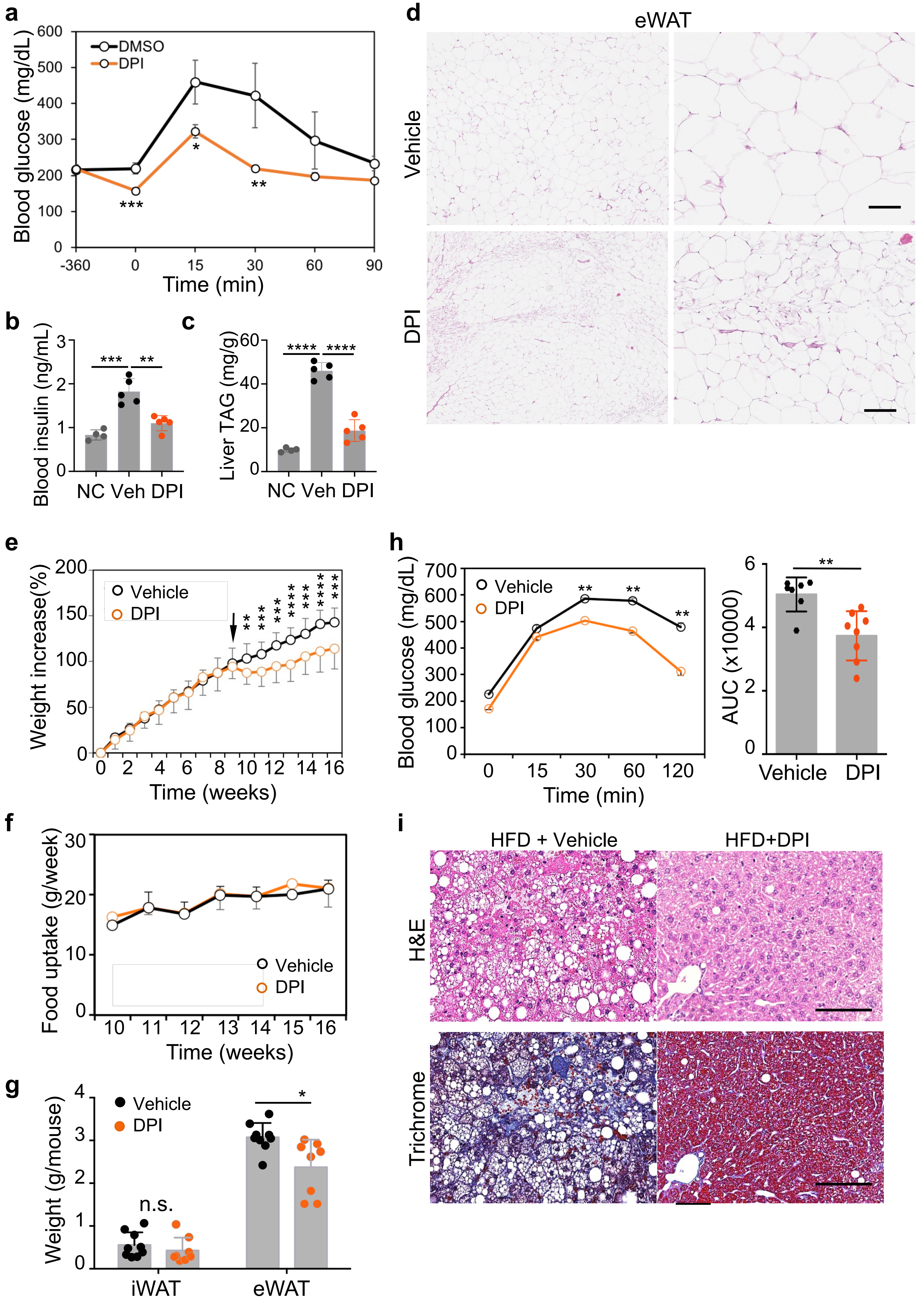

a
DMSO
DPI
Blood glucose (mg/dL)
Time (min)
b
Blood insulin (ng/mL)
NC Veh DPI
c
Liver TAG (mg/g)
NC Veh DPI
d
eWAT
Vehicle
DPI
e
Weight increase(%)
Vehicle
DPI
Time (weeks)
f
Food uptake (g/week)
Vehicle
DPI
Time (weeks)
g
Weight (g/mouse)
Vehicle
DPI
n.s.
iWAT
eWAT
h
Blood glucose (mg/dL)
Vehicle
DPI
Time (min)
AUC (x10000)
Vehicle
DPI
i
HFD + Vehicle
HFD+DPI
H&E
Trichrome

Supplementary Fig. 6. DPI inhibits HFD-induced obesity and liver pathogenesis.
a. C57BL/6 mice at 10 weeks of age were given a single injection of DPI (2 mg/kg) intraperitoneally. Six hrs later (-360 min), mice were injected intraperitoneally with glucose (1 g/kg). Blood glucose levels were monitored at the indicated time. Data are presented as the mean ± sd with 5 mice per group. **b-c.** The blood (plasma) insulin levels and the TAG levels in the liver tissue were quantified by colorimetric assay from mice in Fig. 5a 8 weeks post HFD feeding. **d.** H&E staining of eWAT tissues from mice treated with vehicle or DPI in Fig. 5a. Scale, 100μm. **e-i.** Male B6 mice at 5 weeks of age were fed with HFD for a total of 16 weeks. Nine weeks after HFD (arrow), the mice were dosed with either vehicle or DPI (2 mg/kg) every 5 days for 5 weeks with a total of 7 doses. The weight (**e**) and food consumption (**f**) were monitored weekly. Data are presented as the mean ± sd from two independent experiments with 9-10 mice per group. **g**. Fasting glucose assay. At week 15 plus 3 days, mice from **e** were starved overnight (12~16 hrs) with only water. Glucose (1 g/kg) was injected intraperitoneally and blood glucose levels were measured at the indicated time. AUC were calculated for statistics (right panel). **h**. The weights of eWAT and iWAT after 16 weeks on HFD. **i**. Comparison of H&E and trichrome staining of liver sections from HFD mice (16 weeks) treated with vehicle or DPI. Shown are representative H&E staining from one mouse per group from a. Scale bar: 100 μm. P values were calculated by the student's t-test. * P <0.05, ** P <0.01, *** P <0.001. n.s. not significant.

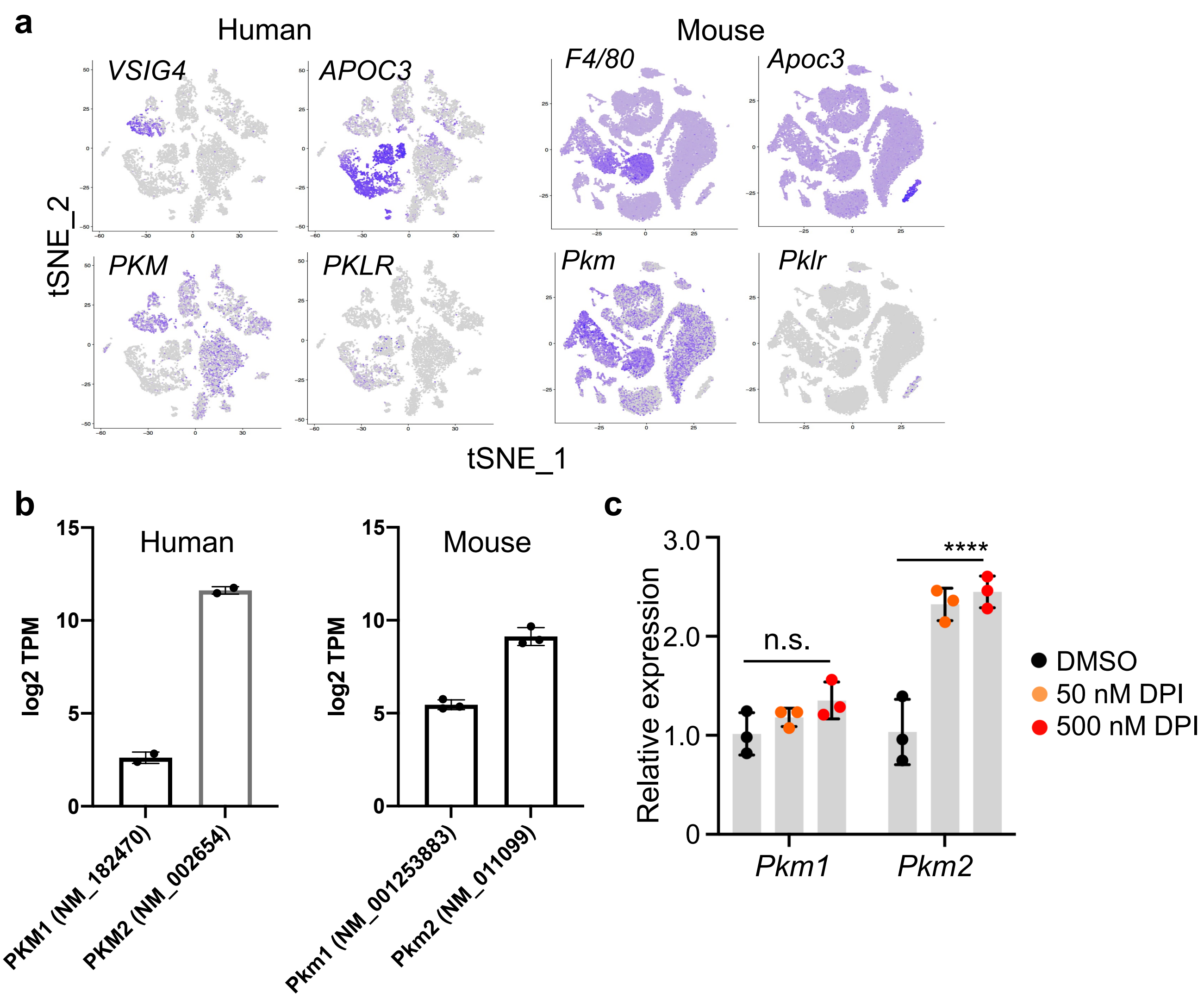

Supplementary Fig. 7. Expression of PKM2 and PKM1 in human and mouse Kupffer cells and hepatocytes. a, scRNAseq data from normal human liver (Aizarani et al. 2019) and mouse liver (Xiong et al. 2019) were reanalyzed for expression of PKM2 and PKM1 as well as markers of macrophages (VSIG4 or F4/80) and hepatocytes (APOC3 or Apoc3) by tSNE plotting. **b**, The expression of PKM1 and PKM2 isoforms in human primary macrophage and mouse Kupffer cells. The raw accounts of each isoform in each sample were calculated with bowtie2 and RSEM (see materials and methods) from human primary macrophages (Hu et al. 2021) and mouse Kupffer cells (Fig. 6 in this study). Expression of PKM1 and PKM2 (log2 TPM) were shown. **c**. The expression of PKM1 and PKM2 isoforms in ImKCs treated with DPI. ImKCs were treated with DMSO or 50 nM or 500 nM DPI for 24 hrs. The transcript levels of *Pkm1* or *Pkm2* were quantified by real-time qPCR. Data were normalized to beta-actin and then to DMSO from three independent experiments with 2 biological replicates for each experiment. **** P<0.0001 by t-test.

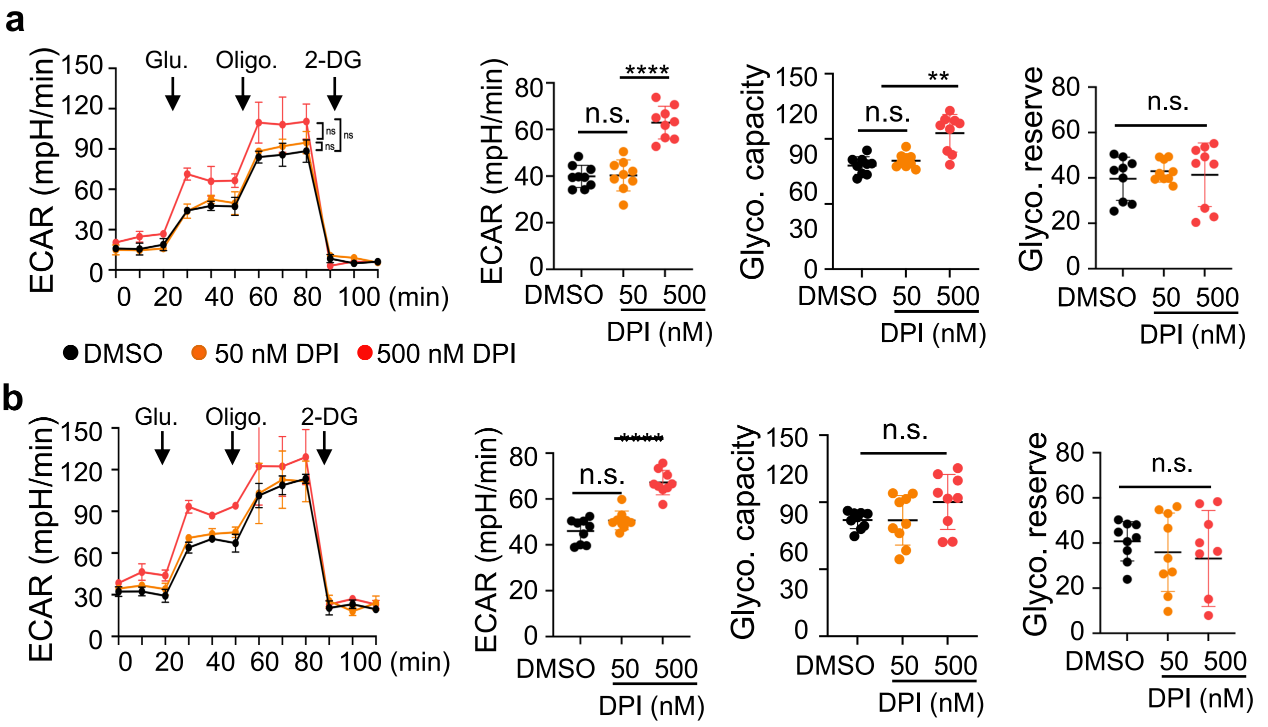

Supplementary Fig. 8. DPI effects on hepatocytes and adipocytes. a. The effects of DPI on ECAR in mouse primary hepatocytes. Hepatocytes were freshly isolated from B6 mouse liver and ECAR were measured with or without DPI (50 and 500 nM) by Seahorse analyzer. Data are presented as the mean ± sd (n=9) from three independent experiments. **b.** The effects of DPI on ECAR in mouse adipocytes. Adipocytes were differentiated from 3T3-L1 cells with MDI (methylisobutylxanthine, dexamethasone, insulin) induction medium and followed with insulin medium. ECAR were measured with or without DPI (50 and 500 nM) by Seahorse analyzer. Data are presented as the mean ± sd from three independent experiments (n=9 biological replicates). P values were calculated by the student's t-test. ** P <0.01, **** P<0.0001. n.s. not significant.

a

P-values	HFD flox Veh. vs DPI	HFD KO Veh. vs DPI	HFD + DPI flox vs KO
Week 1	0.9770	0.9816	0.5569
Week 2	0.2010	0.3094	0.5040
Week 3	**0.0118**	0.9736	**0.0256**
Week 4	**0.0505**	0.4184	0.1254
Week 5	**0.0509**	0.5022	0.2529
Week 6	**0.0266**	0.5745	**0.0608**
Week 7	**0.0234**	0.8116	**0.0601**
Week 8	**0.0257**	0.8333	**0.0077**

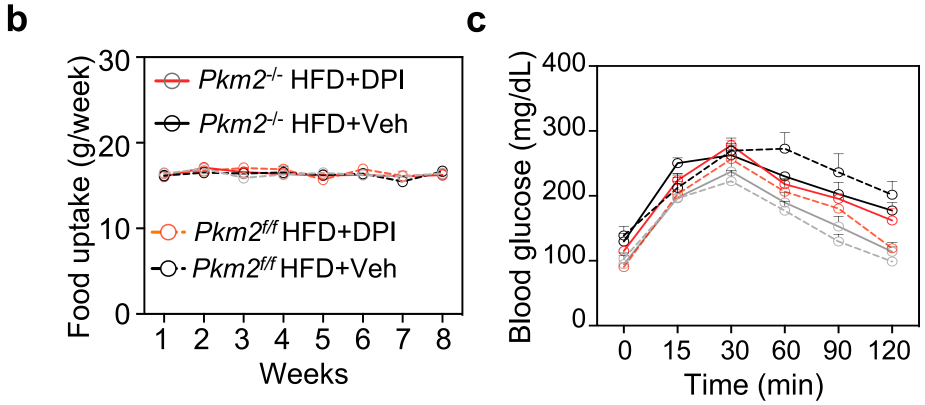

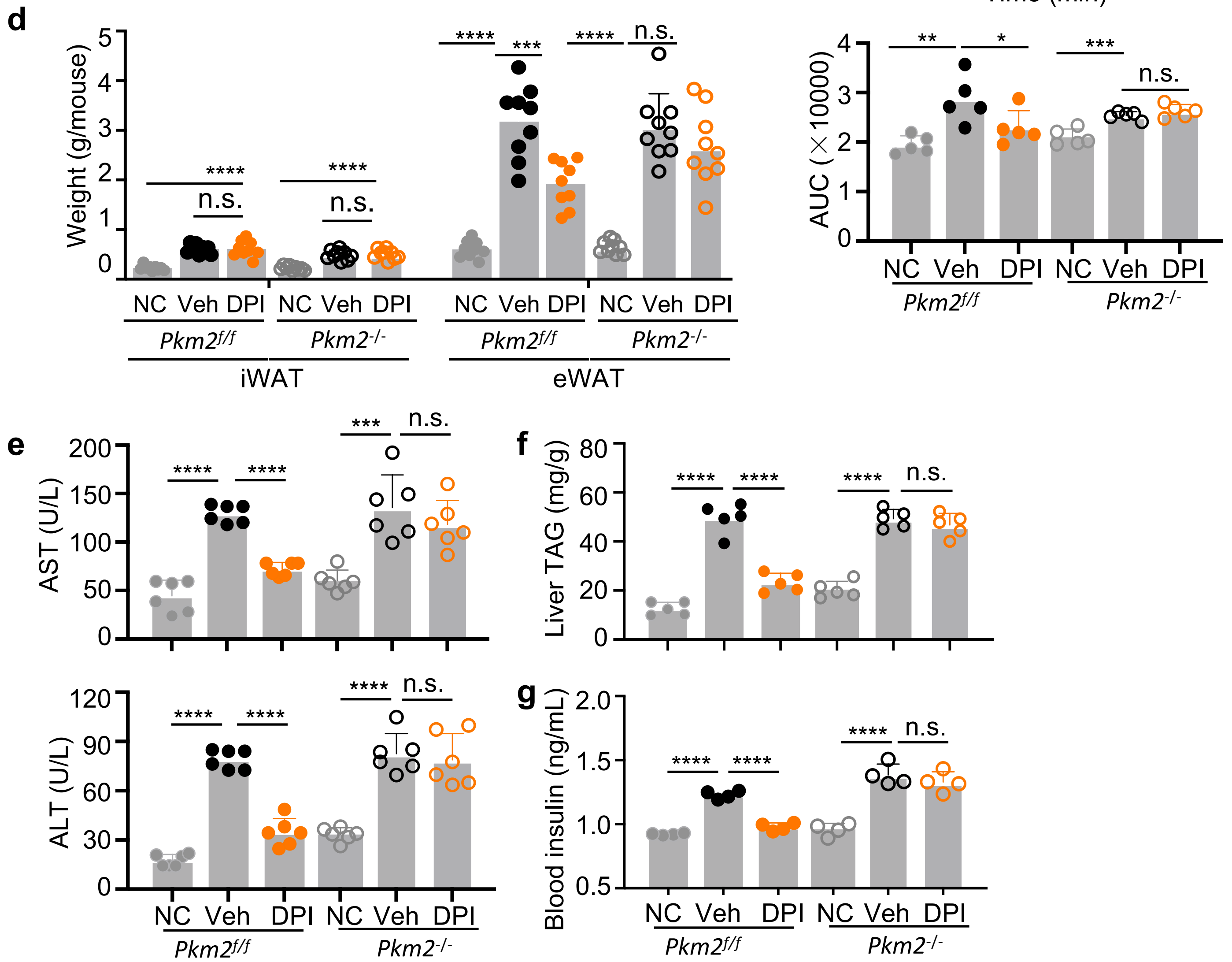

Supplementary Fig. 9. Effects of DPI on *Pkm2* $^{f/f}$ and *Pkm2* $^{-/-}$ mice. Male KC-specific *Pkm2* $^{-/-}$ mice and control *Pkm2* $^{f/f}$ mice at the age of 5 weeks were fed with HFD or NC for a total of 8 weeks. Three weeks after HFD, the mice were given either DPI (2 mg/kg) or vehicle every 5 days for a total of 6 doses. **a**. The statistics of body weight changes between treatment groups among *Pkm2* $^{f/f}$ (flox) and *Pkm2* $^{-/-}$ (KO) mice as shown in Fig.5**g**. **b**. Weekly food consumption. **c.** Fasting glucose assay. At 7 weeks plus 3 days, mice were starved overnight (12~16 hrs) with only water. Glucose (1 g/kg) was injected intraperitoneally and blood glucose levels were monitored at the indicated time. AUC (down panel) were calculated for statistics. **d**. The weights of eWAT and iWAT after 8 weeks on HFD. **e**. Serum levels of AST and ALT after 8 weeks on HDF. **f**. Liver ATG levels after 8 weeks on HDF. **g.** The blood (plasma) insulin levels after 8 weeks on HDF. Shown are representative data from two independent experiments with 5~6 mice per group. P values were calculated by the student's t-test. * P<0.05, ** P <0.01, **** P<0.005. n.s. not significant.

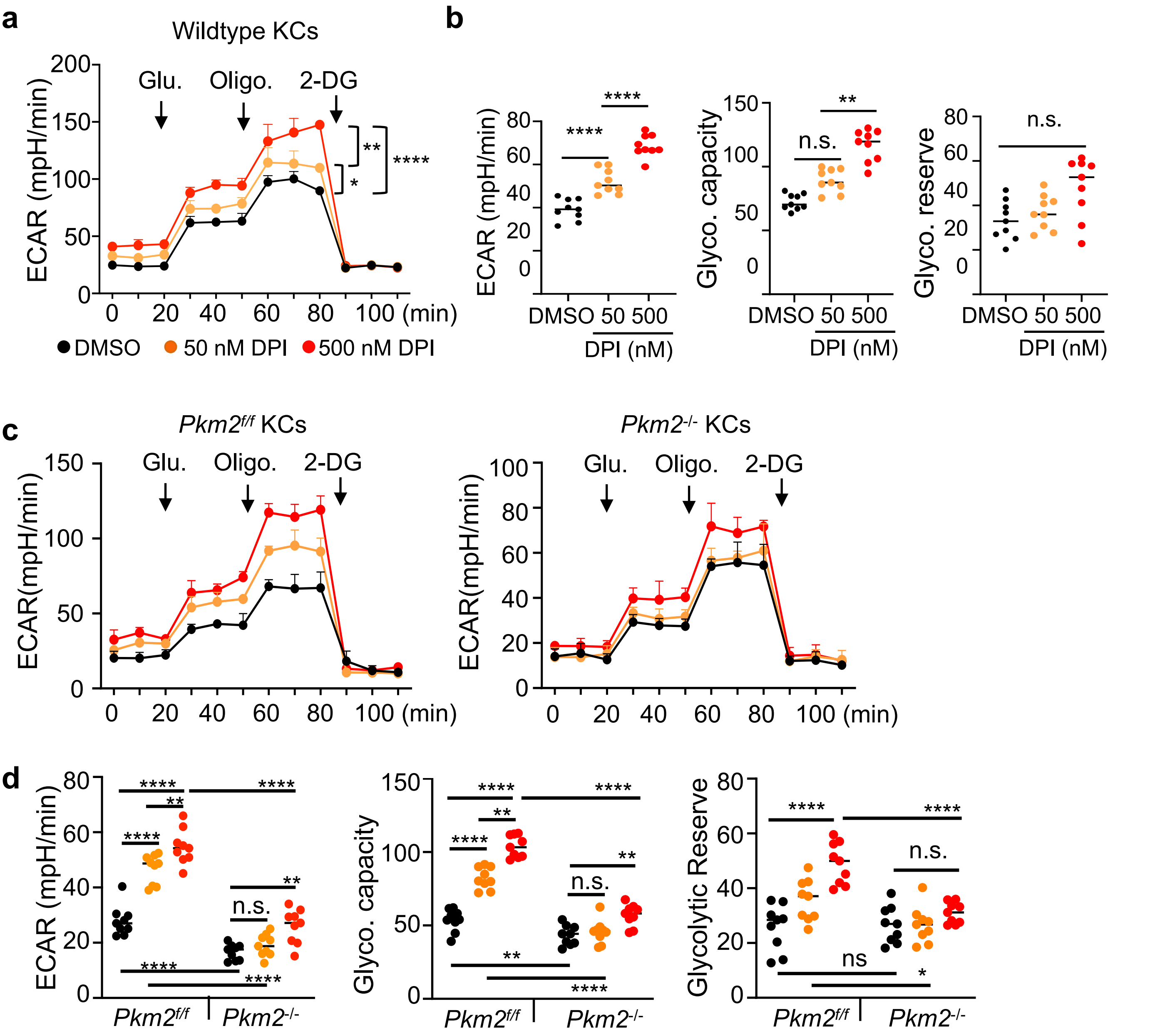

Supplementary Fig. 10. Effects of DPI on wildtype, *Pkm2*$^{f/f}$ and *Pkm2*$^{-/-}$ Kupffer cells. F4/80^{+} Kupffer cells were freshly isolated from the livers of B6 (**a-b**), *Pkm2*$^{f/f}$, KC-specific *Pkm2*$^{-/-}$ mice (**c-d**) and ECAR were measured with or without DPI (50 and 500 nM) by Seahorse analyzer. Specific parameters for glycolytic capacity and glycolytic reserve were calculated and summarized based on three independent experiments. Data are presented as the mean ± sd (n=9 biological replicates). Each symbol in **d** represents one mouse. P values were calculated by the student's t-test. * P<0.05, ** P <0.01, **** P<0.0001. n.s. not significant.

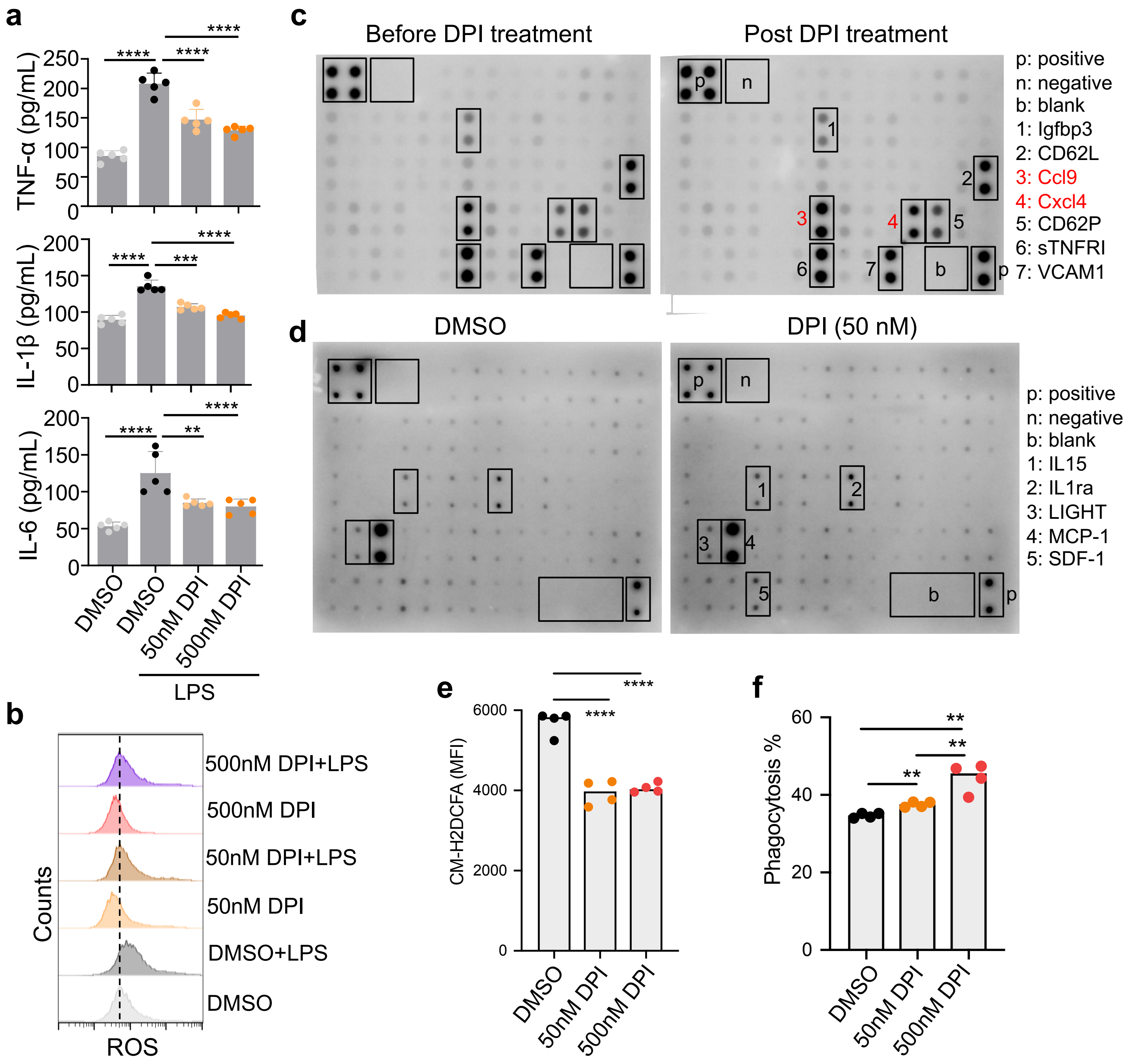

Supplementary Fig. 11. DPI inhibited production of inflammatory cytokines and ROS. a-b. F4/80$^+$ KCs were freshly isolated from B6 mouse livers and treated with DMSO, 50 or 500 nM DPI for 24 hrs. Cells were then incubated with or without 100ng/mL LPS for 6 hrs in the presence of 5 μM CM-H2DCFDA. Cytokines TNF-α, IL-1β and IL-6 in the supernatant were quantified by ELISA (**a**). Total ROS were quantified by flow cytometry. Shown are representative histograms of basal and LPS-induced ROS levels in KCs (**b**). **c**. B6 mice (n=4) were dosed with 2 mg/kg DPI i.p. and plasma were collected before dosing and 48 hrs after dosing. Equal amount of plasma from four mice at the same time-point was mixed and applied to the mouse cytokine antibody array (62 targets with duplicate spots for each on the film). Positive, negative, blank controls and significantly expressed proteins are labeled and listed. Ccl9 and Cxcl4 appear to be upregulated in plasma of DPI-treated mice. **d.** Monocytes were purified from PBMCs from four healthy donors and cultured in the presence of M-CSF to generate hMDMs. hMDMs were treated with DMSO or 50 nM DPI for 24 hrs**.** Equal amount of culture supernatant from four donors was mixed and applied to the human cytokine antibody array (60 targets with duplicate spots for each on the film). Positive, negative, blank controls and significantly expressed proteins are labeled and listed. **e**. hMDMs were treated with 50 or 500 nM DPI for 24 hrs and then incubated with 5 μM CM-H2DCFDA and total ROS were quantified by flow cytometry. **f.** hMDMs were treated with 50 or 500 nM DPI for 24 hrs and then incubated with 15 μg/mL pHrodo Green *E. coli* bioparticles for 1 hr. Phagocytosis was quantified by flow cytometry. Each dot represents hMDMs from one mouse/donor. P values were calculated by the student's t-test. * P<0.05, ** P <0.01, **** P<0.0001.

a

Donor Liver	Steatosis	Cholestasis	fibrosis	lymphocyte infltration
Healthy 1	<5%	no	no	no
Healthy 2	<5%	<10%	no	mild
Healthy 3	<5%	no	no	no
NFALD 1	~15% bullous	~5%	no	mild
NFALD 2	~10% bullous	~5%	mild	no
NFALD 3	~40% bullous	no	mild	no

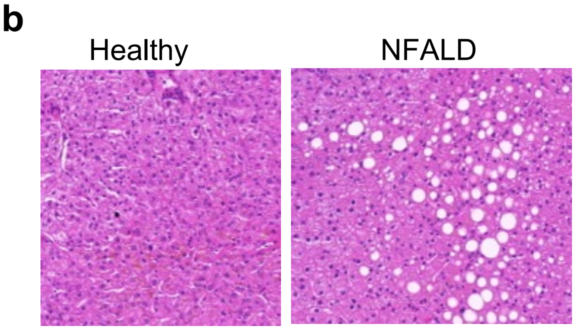

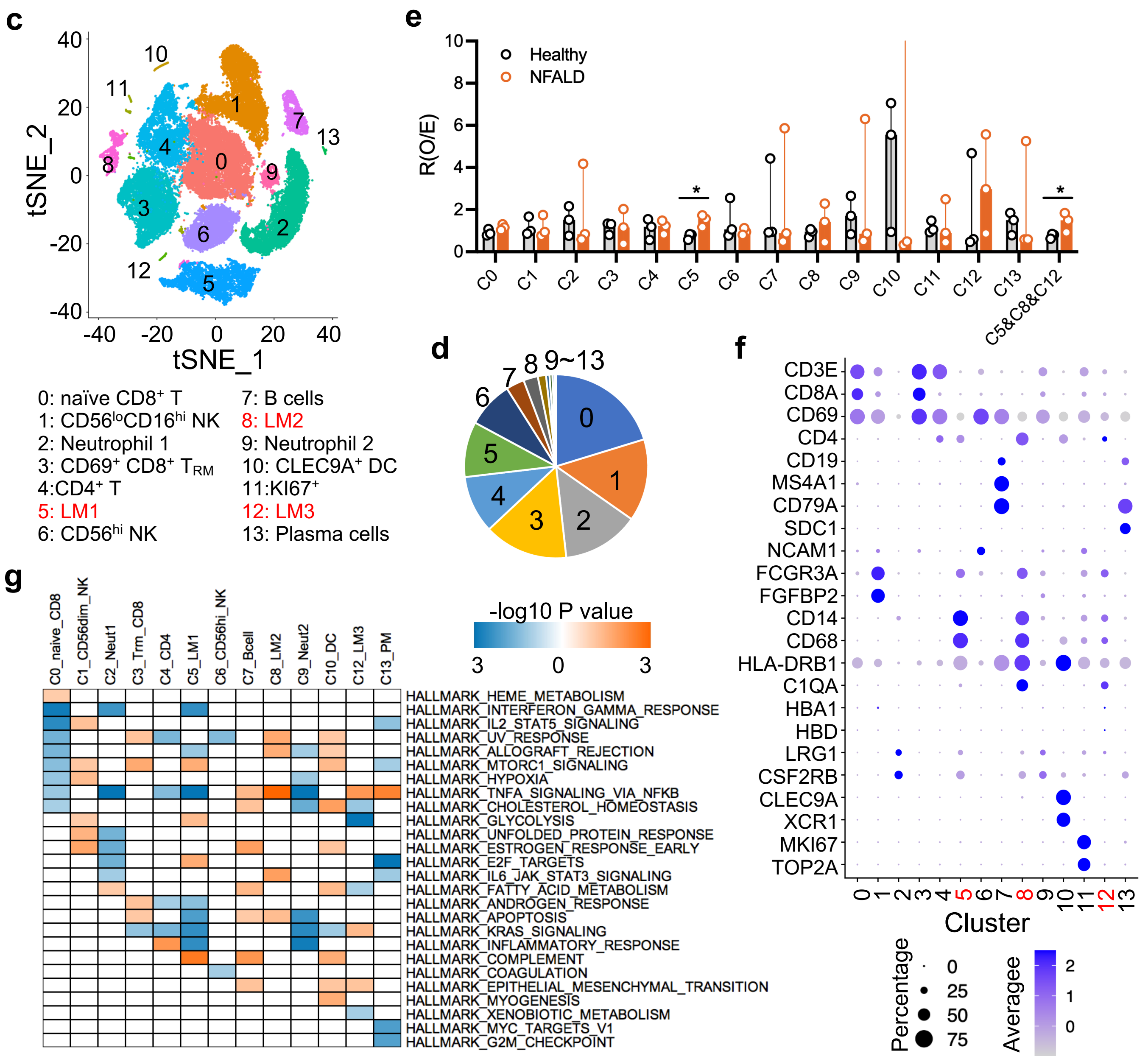

Supplementary Fig. 12. Single cell RNAseq analysis of immune cells from biopsies of healthy and NAFLD human livers. a-b, Clinic information of 3 healthy and 3 NAFLD liver biopsies (a) and representative of HE staining (b) of liver biopsies of healthy and NFALD. **c-f**. tSNE plot of a total 47,724 $CD45^+$ immune cells sequenced (**c**). 14 clusters are identified, the overall cell fractions (**d**) for each cluster and the ratio of observed to expected number of cells for each cluster of each donor were calculated by Ro/e chi-square test (**e**). Statistics between NAFLD and healthy samples were calculated by Wilcoxon signed rank test. * P<0.05. Each cluster was annotated based on the expression of typical markers as T and B cells, NK cells, macrophages, neutrophils and dendritic cells as shown by dot plotting (**f**). **g**. GSEA of gene expression of each cluster with the genes list preranked by fold changes between NFALD patients and healthy donors. Hallmark terms were enriched with P values <0.05 were shown. Orange: positive enrichment. Blue: negative enrichment.

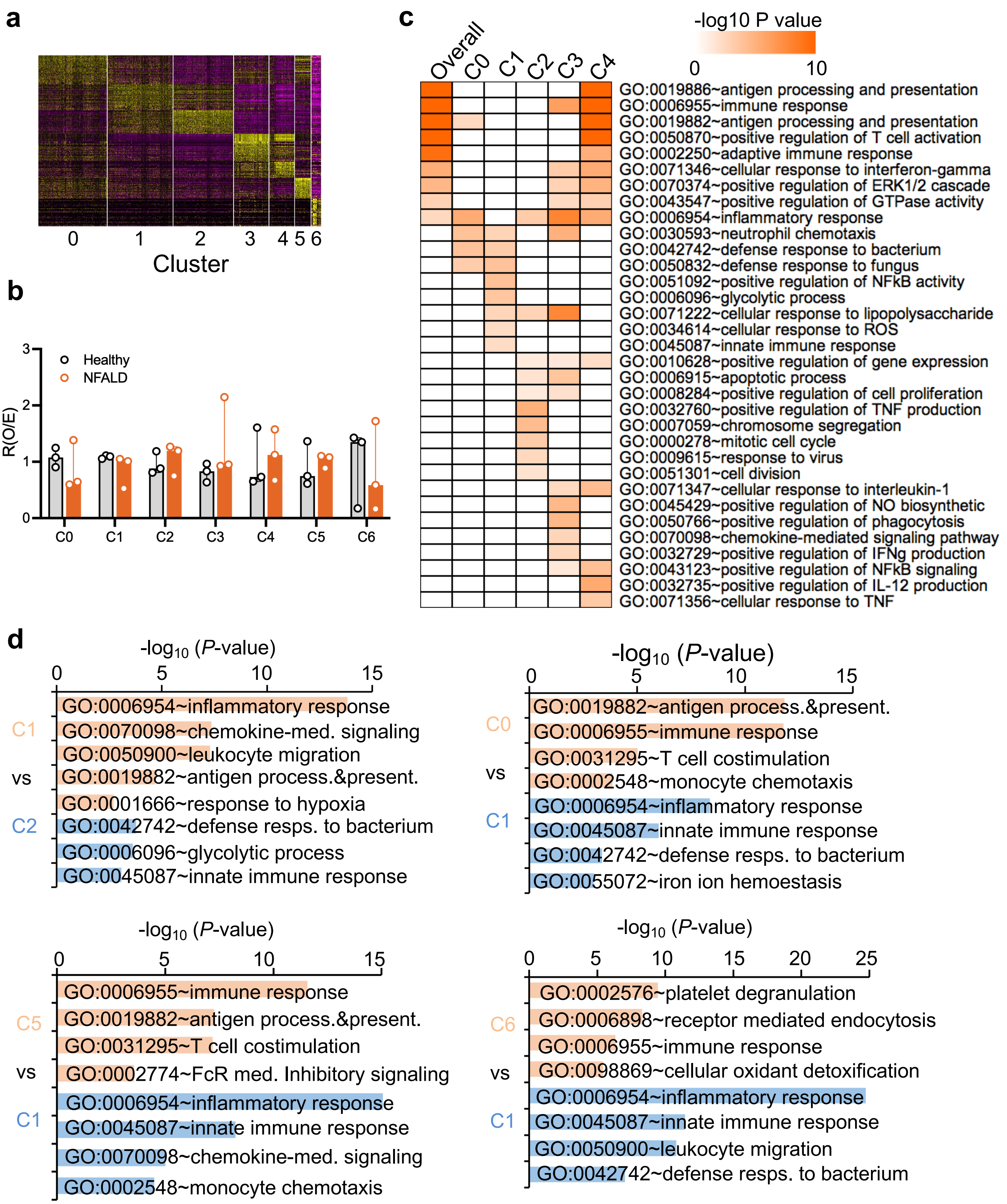

Supplementary Fig. 13. GO enrichment analysis of DEGs of different liver macrophage subpopulations. a. Heatmap of top 10 differentially expressed genes for each cluster. **b.** The ratio of observed to expected number of cells for each cluster of each donor were calculated by Ro/e chi-square test and plotted. **c**. GO enrichment of DEGs up-regulated in NFALD comparing to healthy donor for each cluster. The FindMarkers function was set with min.fct=0.25 and logfc.threshold=0.1. Shown are the heatmap of enriched GO terms with P values<0.05. **d**. GO enrichment analysis of DEGs between different clusters. DEGs as indicated were identified by the FindMarkers function with setting min.fct=0.25 and logfc.threshold=0.25. Shown are the selected top GO terms and P values. Blue: down-regulated, orange: up-regulated.
