# Supplementary material for: Activation of GPR3-β-arrestin2-PKM2 pathway in Kupffer cells stimulates glycolysis and inhibits obesity and liver pathogenesis
Source: Nat Commun. 2024 Jan 27;15:807. doi: 10.1038/s41467-024-45167-5 (PMC10821868; doi:10.1038/s41467-024-45167-5)
Supplement: Supplementary file 3 — Description of Additional Supplementary Files [file 41467_2024_45167_MOESM3_ESM.pdf]

Description of Additional Supplementary Files

Supplementary Data 1
DEGs of mKCs between HFD and NC and between DPI and vehicle.

Supplementary Data 2
PreRanked gene list for GSEA.

Supplementary Data 3
The expression changes of hKCs treated with DPI. The DEG with logFC, LR and P values were computed by edgeR program with the GLM model and exact test test.

Supplementary Data 4
The raw data of metabolic profiling of ImKC response to DPI.

Supplementary Data 5
The raw data of metabolic profiling of *Gpr3*$^{-/-}$ ImKC response to DPI.
