# Supplementary material for: Activation of GPR3-β-arrestin2-PKM2 pathway in Kupffer cells stimulates glycolysis and inhibits obesity and liver pathogenesis
Source: Nat Commun. 2024 Jan 27;15:807. doi: 10.1038/s41467-024-45167-5 (PMC10821868; doi:10.1038/s41467-024-45167-5)
Supplement: Supplementary file 5 — Reporting Summary [file 41467_2024_45167_MOESM5_ESM.pdf]

nature portfolio

Corresponding author(s): Guangan Hu
Last updated by author(s): 1/10/2024

Reporting Summary

Nature Portfolio wishes to improve the reproducibility of the work that we publish. This form provides structure for consistency and transparency in reporting. For further information on Nature Portfolio policies, see our Editorial Policies and the Editorial Policy Checklist.

Statistics

For all statistical analyses, confirm that the following items are present in the figure legend, table legend, main text, or Methods section.

n/a	Confirmed	
☐	☒	The exact sample size (n) for each experimental group/condition, given as a discrete number and unit of measurement
☐	☒	A statement on whether measurements were taken from distinct samples or whether the same sample was measured repeatedly
☐	☒	The statistical test(s) used AND whether they are one- or two-sided<br>*Only common tests should be described solely by name; describe more complex techniques in the Methods section.*
☒	☐	A description of all covariates tested
☒	☐	A description of any assumptions or corrections, such as tests of normality and adjustment for multiple comparisons
☐	☒	A full description of the statistical parameters including central tendency (e.g. means) or other basic estimates (e.g. regression coefficient) AND variation (e.g. standard deviation) or associated estimates of uncertainty (e.g. confidence intervals)
☐	☒	For null hypothesis testing, the test statistic (e.g. F, t, r) with confidence intervals, effect sizes, degrees of freedom and P value noted<br>*Give P values as exact values whenever suitable.*
☒	☐	For Bayesian analysis, information on the choice of priors and Markov chain Monte Carlo settings
☒	☐	For hierarchical and complex designs, identification of the appropriate level for tests and full reporting of outcomes
☒	☐	Estimates of effect sizes (e.g. Cohen's d, Pearson's r), indicating how they were calculated

Our web collection on statistics for biologists contains articles on many of the points above.

Software and code

Policy information about availability of computer code

Data collection	Detailed in the Methods section.
Data analysis	bowtie2 2.2.3, RSEM 1.2.15, Cell ranger 3.1.0, GSEA 4.0.1, MeV 4.8.1, R version (3.5.3) with pakages of Seurat 3.0, Slingshot 2.4.0 and edgeR 3.24.3, Image J 1.53t, Graphpad prism 8.2.0, Axio Vision software, Aperio ImageScope 12.3.3, FlowJo 8.8.7, XFe96 software (Agilent).

For manuscripts utilizing custom algorithms or software that are central to the research but not yet described in published literature, software must be made available to editors and reviewers. We strongly encourage code deposition in a community repository (e.g. GitHub). See the Nature Portfolio guidelines for submitting code & software for further information.

Data

Policy information about availability of data

All manuscripts must include a data availability statement. This statement should provide the following information, where applicable:

- Accession codes, unique identifiers, or web links for publicly available datasets
- A description of any restrictions on data availability
- For clinical datasets or third party data, please ensure that the statement adheres to our policy

Raw RNA-seq data are deposited in the database of Gene Expression Omnibus with accession IDs: GSE186329. The metabolic raw data and DEGs are provided as Supplementary Tables. All other data that support the findings of this study are available from the corresponding authors upon reasonable request. Source data are provided with this paper as a source data file.

Research involving human participants, their data, or biological material

Policy information about studies with human participants or human data. See also policy information about sex, gender (identity/presentation), and sexual orientation and race, ethnicity and racism.

Reporting on sex and gender	Human livers were from 5 NAFLD (2 females and 3 males) and 5 healthy donors (3 females and 2 males).
Reporting on race, ethnicity, or other socially relevant groupings	All human biopsies are Asian Chinese. Commercial human PBMCs don't have information.
Population characteristics	Human peripheral blood were purchased from Red blood components LLC, and subjects were defined healthy status. Human liver biopsies were from livers from deceased donors procured for liver transplantation.
Recruitment	None.
Ethics oversight	Biopsies were from livers deemed unacceptable for liver transplantation. Biopsies were collected with the approval from The Ethics Committee of The First Hospital of Jilin University. Written informed consent was obtained from the legal representative of each donor. Protocols associated with human fresh blood are approved by IRB at MIT.

Note that full information on the approval of the study protocol must also be provided in the manuscript.

Field-specific reporting

Please select the one below that is the best fit for your research. If you are not sure, read the appropriate sections before making your selection.

☒ Life sciences ☐ Behavioural & social sciences ☐ Ecological, evolutionary & environmental sciences

For a reference copy of the document with all sections, see nature.com/documents/nr-reporting-summary-flat.pdf

Life sciences study design

All studies must disclose on these points even when the disclosure is negative.

Sample size	Sample sizes were determined according to previous data published by us (Ref.33 and Hu et al. 2019, 45:563 eBiomMedicine) and others using similar biological samples and techniques to detect the significant difference (Chen et al. 2018, 9:873, Nature Communications). All tests were performed with n>=2 independent experiments. In vivo experiments included data from n=4-12 mice for each group and presented as mean with deviation in the graphs and figure legends. Sample sizes and statistical data are reported in figure legends.
Data exclusions	No data were excluded.
Replication	Data reproducibility was confirmed by independent experiments as indicated in the figure legends or methods.
Randomization	All animals were randomly assigned to different groups for treatments and control as well. Cell culture experiments with cell lines were handled the same way. Bone marrow derived macrophages from one single mouse was taken as one independent sample. Human monocytes were isolated from human PBMC which randomly purchased from the commercial vendor and randomly allocated into groups. Human biopsies were collected randomly based on the availability and tissue weight for tissue procession and cell purification.
Blinding	The investigators were not blinded to all conditions as they were responsible for both experimental design and data collection. The mouse weight and food intake were measured by one technician without knowing the information of mouse group. No subjective assessments were made.

Reporting for specific materials, systems and methods

We require information from authors about some types of materials, experimental systems and methods used in many studies. Here, indicate whether each material, system or method listed is relevant to your study. If you are not sure if a list item applies to your research, read the appropriate section before selecting a response.

Materials & experimental systems

n/a	Involved in the study
	☒ Antibodies
	☒ Eukaryotic cell lines
☒	Palaeontology and archaeology
	☒ Animals and other organisms
☒	Clinical data
☒	Dual use research of concern
☒	Plants

Methods

n/a	Involved in the study
☒	ChIP-seq
	☒ Flow cytometry
☒	MRI-based neuroimaging

Antibodies

Antibodies used

Flow cytometry : antibodies specific for CD11b (M1/70, #101228), F4/80 (BM8, #123116), CD45.2 (104,#109838), and Gr-1 (RB6-8C5, #108406) , anti-human CD45 (#304016) and anti-human CD14 (#301804) from Biolegend. Anti-PKM2 (1C11C7, #60268) were purchased from Thermo Fisher Scientific. Anti-GPR3 (#sc390276) was from Santa Cruz Biotechnology. Anti--arrestin2 (#3857), Glycolysis Antibody Sampler Kit (#8337 and #12866), anti-Lamin B1 (#12586), anti-β-tubulin (#56739) or anti-β-actin (#5125) and anti-FLAG (#) were from Cell Signaling Technology. Human and mouse antibody arrays were from Abcam (#Ab169817 and #Ab133995).

Validation

All commercial antibodies were validated by the manufacture and commonly used in our lab. Information about host species, reactivity, and applications are freely available from manufacturer's websites.

Antibodies for WB were validated by the companies and users. Anti-PKM2 (1C11C7), #60268, ThermoFisher, 1:1000 for WB, validated by the companies and by users. https://www.thermofisher.com/antibody/product/PKM2-Antibody-clone-1C11C7-Monoclonal/60268-1-IG. Anti-GPR3, #SC390276, Santa Cruz Biotechnology. 1:500 for WB, validated by the companies and by users. https://www.scbt.com/p/gpr3-antibody-b-5. Anti-β-tubulin, #56739, Cell Signaling Technology, 1:1000 for WB, validated by the companies and by users. https://www.cellsignal.com/product/productDetail.jsp?productId=56739. anti-β-actin, #5125, Cell Signaling Technology, 1:1000 for WB, validated by the companies and by users. https://www.cellsignal.com/product/productDetail.jsp?productId=5125. Glycolysis Antibody Sampler Kits, #8337 and #12866, Cell Signaling Technology, 1:1000 for WB, validated by the companies and by users. https://www.cellsignal.com/product/productDetail.jsp?productId=8337, https://www.cellsignal.com/product/productDetail.jsp?productId=12866. Anti--arrestin2, #3857, Cell Signaling Technology, 1:1000 for WB, validated by the companies and by users.https://www.cellsignal.com/product/productDetail.jsp?productId=3857. anti-Lamin B1, #12586, Cell Signaling Technology, 1:1000 for WB, validated by the companies and by users.https://www.cellsignal.com/products/primary-antibodies/lamin-b1-d4q4z-rabbit-mab/12586.

For flow cytometry, all antibodies were validated by the companies and by users. anti-CD11b (Biolegend, #101228, 1:100), https://www.biolegend.com/en-us/products/percp-cyanine5-5-anti-mouse-human-cd11b-antibody-4257. Anti-F4/80 (Biolegend, #123116, 1:100), https://www.biolegend.com/en-us/products/apc-anti-mouse-f4-80-antibody-4071. Anti-Gr-1 (Biolegend, #108406, 1:100), https://www.biolegend.com/en-us/products/fitc-anti-mouse-ly-6g-ly-6c-gr-1-antibody-458. Anti-CD45.2 (Biolegend, #109838, 1:100), https://www.biolegend.com/en-us/products/brilliant-violet-510-anti-mouse-cd45-2-antibody-7998. anti-human CD45 (Biolegend,#304016, 1:20), https://www.biolegend.com/en-us/products/pe-cyanine7-anti-human-cd45-antibody-1915. anti-human CD14 (Biolegend, #301804, 1:20), https://www.biolegend.com/en-us/products/fitc-anti-human-cd14-antibody-794

Human and mouse antibody arrays were from Abcam (#Ab169817 and #Ab133995), were validated by the companies and by users.

Eukaryotic cell lines

Policy information about cell lines and Sex and Gender in Research

Cell line source(s)

Immortalized Kupffer cell line (ABI-TC192D, AcceGen), human primary KCs (ABC-TC4369, AcceGen), THP-1 (ATCC TIB-202) and 293T (CRL-3216).

Authentication

The cell lines were not authenticated.

Mycoplasma contamination

Cells were tested for mycoplasma contamination and were negative by PCR.

Commonly misidentified lines (See ICLAC register)

No commonly misidentified lines were used.

Animals and other research organisms

Policy information about studies involving animals; ARRIVE guidelines recommended for reporting animal research, and Sex and Gender in Research

Laboratory animals

C57BL/6 (B6) mice, p47phox-/-, Clec4f-Cre mice were purchased from the Jackson Laboratory and Pkm2flox mice were described in the our publication and housed in specific pathogen-free facilities at MIT under 12-h light dark cycles, controlled temperature (~23 ° C) and 40~50% humidity with free access to food and water.

Wild animals	This study did not involve in wild animals.
Reporting on sex	Male mice were used for HFD experiments. For BMDM in vitro studies, male and females were randomly used.
Field-collected samples	This study did not involve in field-collected samples.
Ethics oversight	All animal studies and procedures are approved by the Massachusetts Institute of Technology's Committee for Animal Care.

Note that full information on the approval of the study protocol must also be provided in the manuscript.

Plants

Seed stocks	None
Novel plant genotypes	None
Authentication	None

Flow Cytometry

Plots

Confirm that:

- [x] The axis labels state the marker and fluorochrome used (e.g. CD4-FITC).
- [x] The axis scales are clearly visible. Include numbers along axes only for bottom left plot of group (a 'group' is an analysis of identical markers).
- [x] All plots are contour plots with outliers or pseudocolor plots.
- [x] A numerical value for number of cells or percentage (with statistics) is provided.

Methodology

Sample preparation	Described in detail in Methods
Instrument	BD FACS Fortessa used for analysis, BD Aria III used for sorting
Software	BD FACSDiva software for collection and FloJo v8.8.7 for analysis
Cell population abundance	human liver immune cells were sorted with anti-CD45 for scRNAseq and human kupffer cells were sorted by staining with anti-CD14 for in vitro treatment by flow cytometry. Mouse kupffer cells were sorted directly from dissociated mouse livers based on F4/80+CD11b+Gr1low or sorted based anti-F4/80 microbeads. Human monocytes were purified from PBMC using the EasySep human monocyte enrichment kit (STEMCELL, #19059) with ~85% purity for in vitro macrophage differentiation.
Gating strategy	Gating strategy was indicated in the figure legends.

- [x] Tick this box to confirm that a figure exemplifying the gating strategy is provided in the Supplementary Information.
